# Morphological Features of Plants on Ash Settling Ponds. Case Study

**DOI:** 10.3390/plants10040616

**Published:** 2021-03-24

**Authors:** Renata Gamrat, Sławomir Stankowski, Anna Jaroszewska

**Affiliations:** 1 Department of Environmental Management, West Pomeranian University of Technology in Szczecin, Słowackiego 17 Street, 71-434 Szczecin, Poland; renata.gamrat@zut.edu.pl; 2 Department of Agroengineering, West Pomeranian University of Technology in Szczecin, Pawła VI Street, 71-459 Szczecin, Poland; slawomir.stankowski@zut.edu.pl

**Keywords:** pioneer species, plant growth, root length, stalk length

## Abstract

Owing to variable water conditions, chemical conditions of water or ash substrate, ash settling ponds belong to anthropogenic objects which do not easily undergo plant succession. However, there are plants exhibiting biological traits allowing colonisation of a substrate characterised by variability in terms of acidity and heavy metal content. The aim of the study was to determine differences in morphology of plants colonising spontaneous surfaces of ash settling ponds with variability moisture level. We identified also differences in morphology of the plants. Identified: *Agrostis stolonifera*, *Atriplex patula*, *Juncus bufonius*, *Phragmites australis*, *Poa pratensis* and *Ranunculus sceleratus*. The obtained results broaden the knowledge on the bioremediation of degraded areas, indicate species that inhabit the surface of ash settlers. Lower water level in ash settling pond I created more favourable conditions for growth of the aboveground parts of plants, and higher waterlevel in ash settling pond II contributed to a more intensive development of the root part of plants. Considering the generative factors and measurement values of the aboveground part of plants, the best adapted species were *Juncus bufonius* and *Atriplex patula*. Due to changing water level in ash settling ponds, the species to be monitored is *Phragmites australis*—most deeply colonising the surface of ash settling ponds.

## 1. Introduction

Plants can colonise almost any habitats, both natural as well as anthropogenic [1,2]. Diversity of flora and its potential to adapt to variable environmental conditions enables expansion even to extremely transformed areas, such as ash settling ponds [3,4]. Owing to constant changes in water conditions and, consequently, chemical composition of water/substrate, these objects do not easily undergo plant succession [5]. In these objects, the area covered by plants predominantly depends on the level of stagnant water: The lower the level, the stronger the colonisation by highly hydrated plants—hydrophilous, followed by less hydrated plants—hygrophilous, and moisture-loving plants—thicket plants [6]. In the studied area, among numerous plant species present on turfed and wooded surfaces around the ash settling ponds under study (located in the area of “Dolna Odra” Power Plant in Nowe Czarnowo, north-western part of Poland), 6 pioneer plant species were identified: *Agrostis stolonifera* L., *Atriplex patula* L., *Juncus bufonius* L., *Phragmites australis* (Cav.) Trin. ex Steud., *Poa pratensis* L. and *Ranunculus sceleratus* L. [4].

*Agrostis stolonifera* (synonym—*Agrostis alba* L.) is a species in the family *Poaceae* (R. Br.) Barnh. common throughout the area of Poland mainly on water shores, wet meadows, pastures as well as dry gravel pits. Tufts of creeping bent predominantly form by rooting of long and frail stolons, less often generatively, that is from small seeds. Owing to very high ecological strength, this is a resistant, easily-rooting perennial plant [7]. *Phragmites australis* (synonym—*Phragmites communis* Trin.) is a cosmopolitan plant in the family *Poaceae* growing on edges of standing or slow moving water. Strongly developed root system holds the substrate together forming underground, submerged or floating stolons of up to 4 m in length [7]. *Poa pratensis* is a low, stoloniferous and loose-tuft of grass in the *Poaceae* family. It creates an even, strong turf with short underground stolons. The early growth of plants and resistance to mechanical damage compared to other species of grasses contribute to the expansion of this plant through the formation of dense turf on the newly formed surfaces, especially on in fertile, fairly moist soils [7]. *Juncus bufonius* in the family *Juncaceae* Juss. is variable in appearance. This is a very resistant cosmopolitan plant which is capable of strongly fixing the substrate with shallow (up to 10 cm deep), yet well-developed, fibrous root system. It is considered a common weed in sandy, sandy-clay and wetland soils on wet water shores, damp forest trails, fields and uncultivated lands [7]. *Ranuculus sceleratus*, the family *Ranunculaceae* Juss., occurs naturally in Poland in hydrated and heavily fouling habitats, i.e., edges of slow moving water bodies and rivers, ponds as well as wet meadows [7]. It effectively reproduces generatively through numerous seeds (there are several dozens of infructescence in one specimen, up to 100 seeds each). It is considered one of the most poisonous plants in Poland. *Atriplex patula* (synonym—*Atriplex patulum* L.) belongs to family *Amaranthaceae* Juss. It is a cosmopolitan species commonly occurring on uncultivated lands, storage areas, gardens, fields and river banks [7]. It spreads easily through small seeds.

Plant cover on ash settling ponds has protective functions as it prevents volatilisation of ash grain due to over-drying of the surface layer resulting from water evaporation from ash settling pond. Immobilisation of volatile ash parts decreases atmospheric pollution found around these anthropogenic objects [8]. Diversity of plant species identified in the study area (encompassing four habitat types: meadow, reed, field and muddy edges of water body), raises the question which factor allowed the colonisation of this anthropogenic and heavily devastated industrial area [9]. Especially considering the fact that the analysed ash settling ponds are small and not particularly sustainable habitats undergoing primary succession of plants [10]. Biometric measurements of the plant species identified in the ash settling ponds were to confirm their broad ecological tolerance to variable physical conditions (water level) as well as chemical conditions of the habitat (water and ash substrate).

The root system of plants, plays an important role in the uptake of water and nutrients, and thus determines the development of the above-ground part of the plant. Thus, a longer biomass of green parts, i.e., stems, leaves) indicates a better (wider/deeper) development of the root system [11]. However, in the experimental plots, the ash substrate contains not only nutrients, but also numerous heavy metals (HMs) [4,12] the presence of which negatively affects the biochemical processes taking place in the plants. This limitation especially applies to the development of seeds, associated with a significant energy expenditure for the plant. The increase in aboveground mass on raw ash is much lower than in contact with other soil additives [13]. The stronger the influence of zonal factors, the more strongly they influence the morphological features of plants. The water in the clarifier is also a stress element, the deficiency of which is one of the main reasons for lowering the growth of above-ground biomass, especially since the amount of water in the clarifier does not match the needs of plants colonizing such an area. In spring, high water level limits the possibility of seed germination, and in summer its limited availability, by increasing the cohesiveness of the ash substrate, contributing to the reduction of the growth of green mass. Other authors also described an important role in the development of aboveground part of plant by the roots system (e.g., [14,15]) dependending on its forms: Bundle system, pile system, stolons or rhizomes [16]. Species with a deep root system develop best on the ash substrate under water stress (warm summer); however, more favorably influenced on the physical condition of the soil has fibrous, branched roots system [13].

The ash substrate is post-production waste, but for the plants colonizing is a soil substrate. Biometric measurements of the roots with ash (width and length) were to indicate the intensity of roots development. Its greater width depended on the specific water conditions, and its length depended on the ash cohesiveness. The stabilization feature of the plant was important in this non-solid medium.

It was assumed the hypothesis that higher water level in ash settling pond No. II, delaying the possibility of germination of plants covering the ash settling ponds by way of ecological succession, will be reflected by lower values of biometric measurements in the above-grounds and undergrounds parts of the pioneer plants present there. It was also assumed that the unfavorable habitat conditions (water movements, HMs) would make it possible to settle this area only by cosmopolitan pioneering species with specific morphological features (long roots system, fast growth rate) allowing the best functioning in this habitat.

The aim of the studies was to identify differences in morphology of the plants (length/width of stalks, leafs, flowers, roots) colonising, through spontaneous plant succession, two active surfaces of ash settling ponds of different hydration level. In the further studies the authors will focused on determining plant species which manifest best capability to act as hyperaccumulators on these anthropogenic industrial objects.

## 2. Results

Regarding the phytosociological classification, the studied plants represented five phytosociological classes and four types of habitats. The meadow species were classified to class *Molinio-Arrhenatheretea* R. Tx. 1937 (*Poa pratensis* and *Agrostis stolonifera*), the species preferring the muddy areas to *Isoëto-Nanojuncetea* Br.-Bl. et R. Tx. 1943 and *Bidentetea tripartiti* R. Tx. Lohm. et Prsg. 1950 (*Juncus bufonius* and *Ranunculus sceleratus*, respectively), reed bed species to *Phragmitetea* R. Tx. et Prsg. 1942 (*Phragmites australis*), and field to *Stellarietea mediae* R. Tx. Lohm. et Prsg. 1950 (*Atriplex patula*). These species belonged to *Poaceae* grass family (3 species), sporadically to *Juncaceae* family, *Amaranthaceae* and *Ranunculaceae* (1 species each).

The measurements conducted on the identified plant species show differences in the analysed morphological features between the two types of ash settling ponds less hydrated (I) and more hydrated (II) (Table 1). The results show shorter length of grass blade or stalks (from 1.2 to 2.1 times) of the specimens from ash settling pond II in comparison with the specimens from ash settling pond I. The results for roots show an inverse relationship (from 1.2 to 1.5 times), with the exception of *Phragmites australis* and *Atriplex patula*. No differences were found with respect to leaves. In two species, generative features were not identified (*Phragmites australis* and *Agrostis stolonifera*).

The comparison of the values of the morphological features demonstrates significant differences in mean values (Table 2) for the analysed species. Significant variation in mean values was also observed in ash settling ponds, with the exception of the maximum roots length. Additionally, a meaning interaction between the species and ash settling ponds was recorded. This means that the reaction with respect to the analysed features was not uniform depending on the hydration level of ash mass in two ash settling ponds. The values of the coefficient of variation (CV%), which is a measure of the accuracy of the experiment, were from 7.5 to 13.6% thus indicating the correctness of the conducted analyses.

The comparison of the plant species under analysis (Table 3) shows a considerable variation of morphological features.

Common reed was found to have the longest grass blade and leaves; however, its roots were among the shortest. Another grass species—*Agrostis stolonifera*, was characterised by a two times higher number of grass blades in a tuft when compared with a typical tuft grass—*Poa pratensis*. The highest length of flower shoots was demonstrated for *Atriplex patula*, whereas the tallest plants, such as *Phragmites australis* or *Agrostis stolonifera*, had no panicles (Table 1 and Table 3).

The analysis of variance determining the effect of ash settling ponds on the morphological traits of the analysed plants showed that in ash settling pond I the plants were found to be taller and characterised by longer leaves and flower shoots. This relationship was not identified with respect to the number of grass blades in a tuft and root length, which were comparable between the two analysed ash settling ponds (Table 4).

The analysis of correlation and regression between the length of the roots and other morphological features of the identified plant species shows that correlation coefficients are different. The analysis between the length of the roots and other morphological features of the identified plant species shows that with an increasing root length in the case of *Poa pratensis* and *Ranunculus sceleratus*, the length of grass blade or stalk tend to decrease. Regarding the length of leaves of *Juncus bufonius* and *Poa pratensis*, the relationship was inverse—with the increasing root length, the length of leaves was found to increase (Table 5).

**Figure 1:**
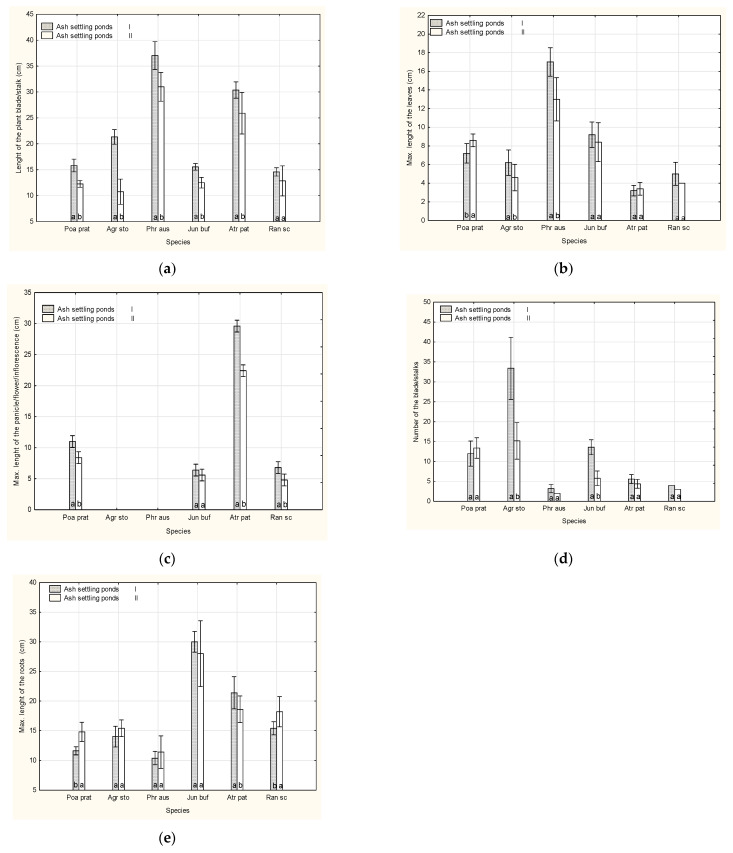
The effect of ash settling pond and species on: Length of the plants blade/stalks (**a**), max. lenght of the leaves (**b**), max. lenght ot the flowers/panicles/inflorescences (**c**), numer of the stalks/blade (**d**), max. lenght of the roots (**e**).

The effect of a given ash settling ponds (I and II) on the morphological features of the six plant species was not uniform. The length of the plants’ blade/stalks showed a non-identical reaction of the flora found on the surfaces of ash settling ponds, with the exception of *Ranunculus sceleratus* (Figure 1a). The remaining species found in ash settling pond I were taller in comparison with their respective species from ash settling pond II. Another trait, i.e., the maximum length of leaves, exhibited differences between grass: *Poa pratensis*, *Agrostis stolonifera* and *Phragmites australis* found in the two ash settling ponds. With respect to green plants (*Atriplex patula*, *Ranunculus sceleratus*), the aforementioned effect of ash settling pond was not found (Figure 1b). As for the length of flower stalks, there were differences in *Atriplex patula*, *Poa pratensis* and *Ranunculus sceleratus*. In *Juncus bufonius*, the distinction in values were not identified, similarly to *Agrostis stolonifera* and *Phragmites australis*—however, owing to lack of panicles (Figure 1c). The number of grass blades or stalks in a tuft or a specimen demonstrated differences only with respect to *Agrostis stolonifera* and *Juncus bufonius*. The remaining species showed comparable values recorded in two ash settling ponds (Figure 1d). The analysis of the length of the roots demonstrated differences in *Poa pratensis* and *Ranunculus sceleratus*—higher values were found for ash settling pond I, and regarding *Atriplex patula* in ash settling pond II (Figure 1e).

The obtained results broaden the knowledge on the bioremediation of degraded areas, indicate species that inhabit the surface of ash settlers. On the area of the ash settling pond, four monocotyledonous plant species were found (*Agrostis stolonifera*, *Juncus bufonius*, *Phragmites australis* and *Poa pratensis*) and two species of dicotyledonous (*Atriplex patula* and *Ranunculus sceleratus*).

Considering the generative factors and measurement values of the aboveground part of plants, the best adapted species were *Juncus bufonius*, followed by *Atriplex patula*, *Poa pratensis* and *Ranunculus sceleratus*. Due to changing water level in ash settling ponds, the species to be monitored is *Phragmites australis*—the only species vegetatively colonising the surface of ash settling ponds most deeply.

## 3. Discussion

Dense turf covering the anthropogenically transformed surfaces accumulates HMs and absorbs odours, therefore it contributes to a decrease in the amount of hazardous substances and improves quality of the atmosphere [17,18]. Power plants with ash settling ponds are located far from densely populated areas. However, fly ash from a dried surface layer of the ash settling pond may pollute the atmosphere at considerable distances [19,20,21,22]. The lack of water above the ash settling ponds contributes to the local air pollution as a result of the volatilization of ash particles, especially in the presence of strong winds [23]. The lower water level of the ash settling ponds—adjusted to the ability of the species to take roots—allowing plants to grow help to reduce of air pollution. This is particularly important in the area of ash settling ponds since the plants found there usually tend to grow close to the ground surface.

Habitat conditions shape the morphological features of plants. From the plant organs, much attention is paid to the root system, on which the intensity of the development of the aboveground part depends. The more intensive this development, the higher the metric parameters: The higher the stems, leaves, flower shoots or inflorescences. Kuczyńska et al. [11] in an experiment using biometric measurements of *Hordeum vulgare* L. of the underground and aboveground parts, showed a positive correlation of these two features at the significance level *p* < 0.01. However, the Lovett-Doust 1981 [24] study found no significant relationship between the development of the underground and the aboveground parts. In the study area, the analysis of the roots length in relation to the stems length indicated a negative correlation in *Poa pratensis* −8.86, *Agrostis stolonifera* −0.77, *Ranunculus repens* −0.75), but positive with the leafs length in *Poa pratensis* 0.75 and *Juncus bufonius* 0.65 (Table 5).

The important role of the roots system in stimulating the growth of the aboveground part under water stress was demonstrated by Streda et al. [14] and Svacina et al. [15] in a field experiment of growing barley and wheat. The analysis of biometric measurements during the ecological succession of the popular *Ranunculus repens* L. species in various meadow habitats (open and post-park) also indicated water conditions as one of the most important factors influencing the possibility of generative reproduction. The water shortage at the end of the growing season caused the new generation of plants to dry up. Even favorable other soil conditions did not change this state—no significance was found at *p* = 0.05 [24].

The plants found in habitats which are extremely transformed in terms of their physical or chemical characteristics, belong to pioneer species and exhibit numerous morphological or physiological adaptive features [25,26,27]. There were differences between the measured values within the species. The smallest differences, for example in the length of the roots system (1.3 times) were found in *Agrostis stolonifera*, and the largest difference (3 times) in *Juncus bufonius* (Table 1). Similar observations regarding the differences in the length of the roots system were shown by Kuczyńska et al. [11] for common barley. The roots length ranged from 10.0 to 20.7 cm, i.e., it was 2.1 times.

The influence of stressful conditions on the intensity of the aboveground growth was demonstrated by Betekhtina et al. [8] noting the greater sensitivity of plant species grown in treeless habitats as opposed to forest habitats. Research by Nawrot et al. [28] showed morphological and structural differences in *Phragmites australis* originating from an environment with less and more HMs pollution. At a higher concentration of HMs, they observed in growth limitation, roots tissue disturbance, roots hair number decrease, and structural alterations in the epidermis and endodermis.

In the study area, plant species adapted to this extreme environment through numerous individual features, such as: A special roots system, adaptation to water conditions or the specificity of generative or vegetative reproduction. Plant species occurring in the analyzed ash settling ponds were characterized by a well-developed roots system in the form of fibrous or longitudinal roots, and they were well stable in the substrate. Perennial species was characterized by high water preferences, i.e., they inhabited the area up to 100% of water capacity, with the exception of *Poa pratensis* (with a water capacity of 85%). Numerous seeds and a long flowering period (from 13 to 22 weeks—Table 6), made it annual plants, possible to exist in this habitat. Particularly gradual maturation of the seeds made it possible to colonise the areas in slightly shallower parts at the water’s edge.

Betekhtina et al. [29] analysing changes in the species composition in ash dump subjected 50 years plant natural succession, indicated permanent cover among others by *Poa pratensis*. This species achieved the highest C/N values (42.5), which testified to the low biological activity of the soils in this habitat, and at the same time about the wide ecological tolerance of this species. In the deeper parts of the ash clarifiers, only the typical rush species—*Phragmites* was found (Table 6). Research by Pandey et al. [30] indicated another species of rushes—*Typha latifolia* L. as one of the most tolerant species in terms of microclimatic and edaphic stresses, and at the same time very effective in sequestering CO_2_ emissions.

Konstantinov et al. [31] pointed to the species as being dominant among grasslands in the areas of this type, which was explained by Dyguś et al. [3] by a very high water-holding capacity of fly ash, which allowed participation in the early stages of the primary succession [32]. However, Tőzsér et al. [33] point to plants from segetal habitats, rather than reed, as being the pioneer plants—such as *Chenopodium album* L. and *Tripleurospermum maritimum* (L.) W. D. J. Koch. The rooting of stolons of *Agrostis stolonifera* (Table 6) allowed fast colonisation of ash settling ponds, especially given the fact that *Agrostis stolonifera* was tolerant to washing [34]. The structure of the substrate itself is a problem in the colonization of ash habitats. Its low bulk density (0.72–0.97 g/cm^3^) makes the ash substrate brittle, and thus makes it difficult to roots plants on this unstable substrate [8].

Small, yet numerous tufts of *Juncus bufonius* found in the analysed ash settling ponds, could, as is argued by Swacha [35], result from spreading numerous seeds (Table 6). In ash lagoons, Kostić et al. [36] identified the presence of three grass species: *Festuca rubra* L. and *Dactylis glomerata* L., *Calamagrostis epigejos* (L.) Roth. and *Oenothera biennis* L.—dry habitat species. Pandey [26] emphasises many traits of the pioneer species, such as: High tolerance to unfavourable conditions, perennial nature, extensive root system, tolerance to high pH values (alkaline reaction (8.3) [8] or to HMs. Among the species capable of colonising these surfaces through natural succession was *Typha latifolia—*a common species of the Polish flora. However, to the areas not so easily undergoing colonisation, Pandey [26] suggests introducing species foreign to the given region, e.g., *Chrysopogon zizanioides* (L.) Roberty, *Cymbopogon flexuosus* (Nees ex Steud.) W. Watson, *C. martini* (Roxb.) W. Watson and *C. winterianus* Jowitt ex Bor. The role of the root system in breaking the compact ash layer and loosening its structure was explained by Uzarowicz et al. [12].

Long-term (25-year) studies of Estonian meadows, using biometric measurements of the identified plant species (98), showed a low significant the relationships of the intensity of growth forms between species with high vegetative mobility (rhizomes, stolons) in relation to species with low vegetative mobility [37]. A similar relationship was found in the study area (Table 6). Species with high vegetative mobility, such as *Agrostis stolonifera* and *Phragmites asutralis*, despite a more versatile roots system compared to other plants species (stoloniferous long roots), have not produced generative parts.

**Figure 2:**
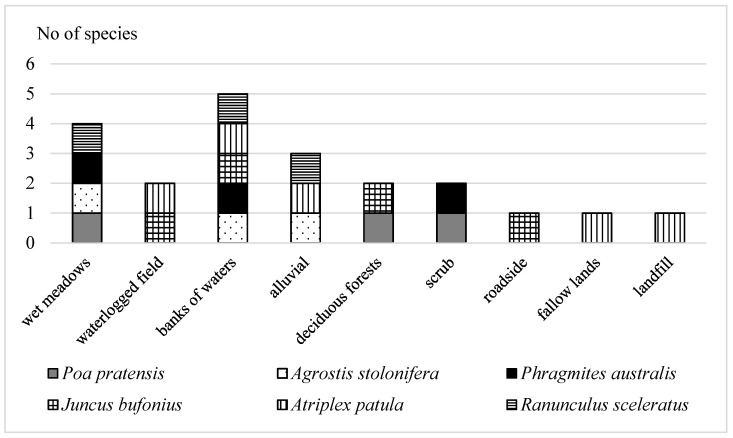
Preferred habitats of studied plant species (according to Plants atlas [7]).

All identified species, according to habitat preference, occurred at the edges of water bodies, with the exception of *Poa pratenis*, though it was also found in abundance on wet meadows (Figure 2). In their studies on plant succession in ash settling ponds, among the 12 species able to spontaneously colonise these anthropogenic surfaces, Kisku et al. [38] identify, among others, *Chenopodium album*, *Solanum nigrum* L. and *Typha angustifolia* L.—common species in the Polish flora. Among the families, grass is the most numerous pioneer species [25], and currently in the analysed area *Agrostis stolonifera*, *Poa pratensis* and *Phragmites australis* show a tendency towards ruderal and segetal habitats, therefore were resistant to variable nutritional and water conditions [27]. The rate of colonisation by *Ranunculus sceleratus* of wetland saturated with ash due to volcanic activity was discussed by Tsuyuzaki [32] in the analysis of flora in the early stages of succession. Another species—*Juncus bufonius*, also present in abundance in the water edge part of the ash settling pond, in the opinion of Swacha [35] was one of the most important species forming the initial stages of plant communities in gravel surfaces of post-mining areas.

The intensification of stress factors limits the development of the plant. In the study area, control values (C) showing the maximum lengths of, for example, parts of shoots, showed large differences with the measured values (P.S.) (Table 1). The largest 12- multiplicity differences were found in *Phragmites australis*, and the smallest (1.8-multiplicity) in *Juncus bufonius*. This was confirmed by studies by Kuczyńska et al. [11] showing a 1.6-multiplicity disproportion between the maximum literature results and the achieved stems length measurements in *Hordeum vulgare* L.

Betekhtina et al. [29] analyzing the species composition in the ash dump, subjected to over 50 years of natural succession, indicated the predominance of grasses from meadow habitats (including, for example, *Poa pratensis*) and nitrophilous grassy (*Calamagrostis epigeios* with a small share of herbs: Meadow (*Molinio-Arrhenatheretea* class: *Pimpinella saxifraga* L., *Achillea millefolium* L.), forest fringe (*Trifolio-Geranietea sanquinei* Th. Müller 1962: *Silene nutans* L.) or dry grassland (*Festuco-Brometea* Br.-Bl. et R.Tx.1943: *Plantago media* L.). However, in the study area plant species belonged mainly to communities strongly associated with water *Phragmitetea*, *Isoëto-Nanojuncetea* and *Bidentetea tripartiti.*

The analysis of species according to their tolerance to the presence of HMS and salinity, performed according Chmiel [39], showed that the studied species, except for *Juncus bufonius*, showed resistance to unfavorable soil conditions, e.g., salinity and/or HMs in the substrate. Due to the wide ecological spectrum and resistance to the above-mentioned stress factors, among the most frequently sown grasses in human degraded areas there were, among others, *Agrostis stolonifera* and *Poa pratensis* [40].

The studies by Falkowski et al. [41] demonstrated that *Agrostis stolonifera* is resistant to pollution with copper, and *Poa pratenis* to pollution with chromium. The studies by Gamrat et al. [4] confirmed only copper phytoaccumulation in *Agrostis stolonifera* as the content of chromium in *Poa pratensis* was among the lowest values. Large area covered with grass species results from the resistance of the species, since, apart from the biological features, the expansion of *Agrostis stolonifera* and *Poa pratensis*, according to Falkowski et al. [41], was made possible due to chemical properties of these plants (cyanogenic glycosides and saponins, respectively).

Apart from HMs constituting the ash, plants succession was hindered by varied substrate acidity values. The identified plant species showed no habitat preference or were found on weakly acidified habitats [7], which allowed colonisation of transformed habitats of low pH values (5.22–6.55 [32]). However, in the area of the analysed ash settling ponds, pH values were high—7.96 [4].

Loss of water through the ash substrate improves the vertical stabilization of plants, but it made rooting more difficult [42]. In the study area, the maximum values of the roots’ measurements compared to their length in the ash settling pond showed the smallest differences in *Phragmites australis* (0.5-multiplicity), and the highest in *Juncus bufonius* (4.7-multiplicity) (Table 1).

Considering the environmental and economic reasons, identification of species spontaneously growing on hydro-sediment are not a threat to other species present around the ash settling ponds. They persist without human interference, therefore the cost of sustaining the composition of undergrowth is lower. Given the knowledge on the negative effects of foreign species on biodiversity, it seems less justified, as stipulated by Woch [6], to introduce foreign species from North America (*Euphorbia marginata* Pursh., *Rudbeckia bicolor* Nutt., *Yucca flaccida* L.) or Asia (*Miscanthus sacchariflorus* (Maxim.) Hack.) to non-active hydro-sedimentation ponds of the Siersza Power Plant in Trzebinia (western Poland). Gupta and Sinha [42] and Maiti and Pandey [43] pointed to the important role—phytostabilization of fly ash settling ponds—near the coal power plant by species naturally colonizing these surfaces. In another publication by Pandey et al. [44] emphasized the importance of the reclamation time. It is not the origin of the species that will determine phytorecultivation, but its speed of decomposition and nutrient release.

## 4. Materials and Methods

### 4.1. Study Area

**Figure 3:**
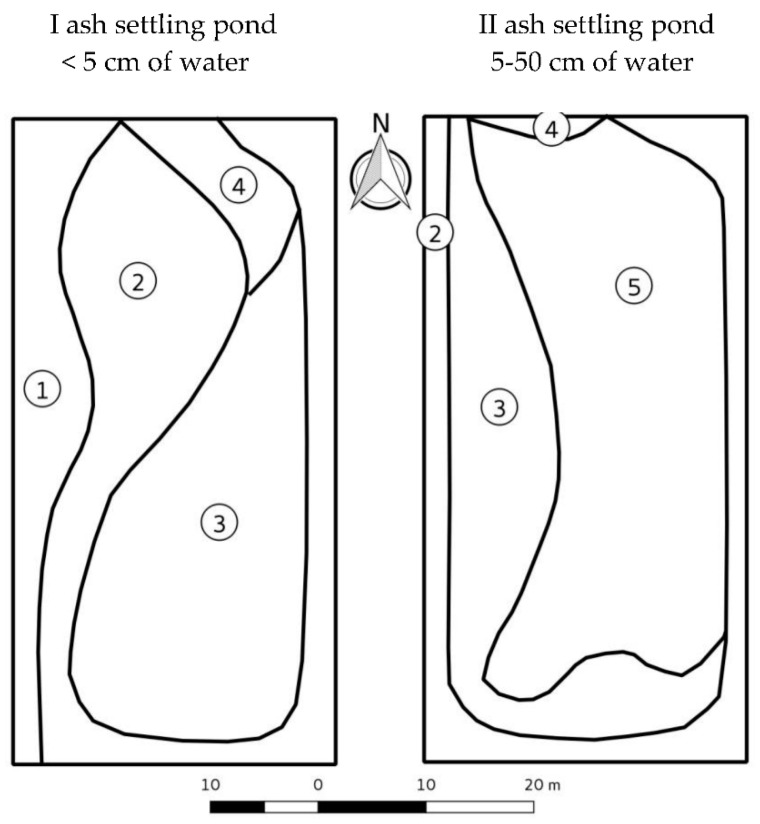
Distribution of dominat species na the area od I and II ash settling ponds (according to Gamrat et al. [4]). 1, *Poa pratensis*, 2, *Juncus bufonius*, 3, *Phragmites australis*, 4, *Ranunculus sceleratus*, 5, no plants.

In the area of “Dolna Odra” Power Plant in Nowe Czarnowo, 53°11′40″ N, 14°29′30″ E (Zachodniopomorskie voivodship, Western Pomerania region, Poland)—the surface of two active of ash settling ponds (Figure 3) was subjected to floristic and chemical analysis, according to the methodology presented by Gamrat et al. [4]. The studied settling ponds showed differences in terms of water surface elevation. The settling ponds no. II was more hydrated, i.e., 50 cm—in the center of the settling surface than the settling ponds no. I, i.e., up to 5 cm—in the center of the settling surface. The ash settling ponds (0.4 ha) were used to store microsphere—a fine fraction of ash resulting from coal combustion in energetic blocks of the power plant.

Some time after the ash settling ponds was flooded and during the gradual drying up, the surface was successively populated by plants. In the first part of the work [4] the species composition of flora was determined in 18 plots that were separated on the surface of the settling ponds, and the chemical composition of the ash substrate and plants was analyzed in terms of the content of selected elements (Table 7).

The present work is an extension of earlier research [4]. At the end of the growing season of 2015 (the beginning of September), random plant specimens of the six plant species were collected from the research site: *Agrostis stolonifera* (abbreviated Agr.sto), *Atriplex patula* (Atr.pat), *Juncus bufonius* (Jun.buf), *Phragmites australis* (Phr.aus), *Poa pratensis* (Poa.pra) and *Ranunculus sceleratus* (Ran.sce). From each of the ash settlers, five clumps/individuals of each plant species were collected.

### 4.2. Features of Ecological Indicators

For the purpose of characterisation of the identified species, they were classified according to family, their main habitats were specified (Figure 2), and their prevalence in Poland was determined [7]. The synanthropodynamic, historical and geographic features were analysed ([39], respectively: Invasive species—very highly-, highly- and poorly-dispersing species colonising habitats other than those suitable, and apophytes—native species colonising secondarily anthropogenically transformed habitats). According to Zarzycki et al. [45] the tolerance of plants to the presence of HMs in soil and the salinity of the habitat was determined. The biological characteristics of plants were also selected, which may affect their greater tolerance to negative environmental factors. Measured flowering length, size and number of seeds, type of root system or life form [7] (Table 6).

### 4.3. Measurements of Morphological Features

**Figure 4:**
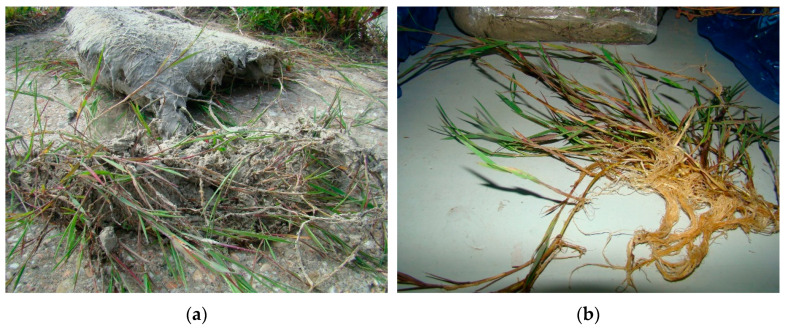
Plant’s material with (**a**) and without (**b**) an ash layer.

For six species on surface of each ash settling pond was taken the random specimen of each plant species. Table 3 shows mean values for five specimens/tufts. Found plant specimens were subjected to morphological measurements the aboveground parts and the belowground of plants: Stalks (length and the number in a tuft or specimen), leaves (maximum and minimum length), flowers (maximum and minimum length) and roots (maximum and minimum length). The measurements were made in cm. The data are summarized in Table 1 as “Private study” (P.S.), in contrast to the literature [7] data marked as control. Control values (C) were recorded from the literature for the maximum and minimum parameters for underground part from plants: Stalks, leaves and flowers. Metric measurements of the roots system were also made with a preserved ash lump formed after being pulled out a plant specimen from the surface of the ash settling pond (Figure 4).

### 4.4. Characteristics of Climatic Conditions

In 2015, mean annual air temperature recorded in the analysed area was 10 °C [46]. According to precipitation classification, the year was considered dry (Table 8), particularly the beginning and the end of the growing season (1—extremely dry, 2—very dry, 3—dry, 4—normal, 5—wet, 6—very wet, 7—extremely wet). Low precipitation values and intense sun exposure were conducive to low water level in ash settling ponds which, consequently, allowed colonisation of the surface by plants.

### 4.5. Statistical Analysis

The results were statistically developed using two-way analysis of variance in a complete randomised design. The number of replications was n = 5. Confidence half-intervals for the comparison of multiple averages were calculated with HSD Tukey test. Simple relationships between the variables were determined with Pearson’s correlation coefficient. Equations of regression lines are given per cases in which the significance of correlation at the minimum level *p* = 0.05 was found [47]. Statistical analysis of the results was carried out using Statistica 10.0 software.

## Figures and Tables

**Table 1:** Biometric measurements [cm] of individual morphological parts of plant species.

Sp.	No	Morphological Parts of Plants																						
		The Above-Ground Part																	The Underground Part					
		Stalks Length							Leafs Length					Flowers Length					Roots					
																			Length		Width		Length	
		No Ash																	with Ash				No Ash	
		C			P.S.				C		P.S.			C		P.S.			P.S.					
		max.	min.		max.	min.	av.		max.		max.	min.		max.		max.	min.		max.		max.		max.	min.
Poa.	I	70	20		24	10	16		20		7	4		20		11	6		7		19		12	5
pra	II	70	20		18	9	12		20		9	4		20		8	4		6		13		15	6
Agr.	I	80	20		24	18	21		10		6	3		5		0	0		5		26		14	8
sto	II	80	20		12	9	10		10		5	3		5		0	0		6		17		15	12
Phr.	I	400	100		39	35	37		50		17	10		40		0	0		17		9		10	7
aus	II	400	100		33	29	31		50		13	9		40		0	0		16		9		11	6
Jun.	I	30	5		17	14	16		10		9	4		0.5		0.5	0.5		7		14		30	10
buf	II	30	5		14	12	13		10		8	4		0.5		0.5	0.5		6		10		28	12
Atr.	I	80	30		41	29	34		4		3	2		60		38	31		10		9		36	20
pat	II	80	30		31	26	27		4		3	1		60		33	26		12		5		34	16
Ran.	I	60	20		21	10	17		6		5	2		30		7	4		8		11		22	8
sce	II	60	20		15	12	13		6		4	2		30		4	3		7		10		19	10

Sp., plant species; No, numbers of ash settling ponds (I, II); C, control; P.S., private study; Stalks, stalks of plant /grass blade; Flowers, flowers/panicles/ inflorescences; Roots, roots/ stolons; Poa.pra, *Poa pratensis*; Agr.sto, *Agrostis stolonifera*; Phr.aus, *Phragmites australis*; Jun.buf, *Juncus bufonius*; Atr.pat, *Atriplex patula*; Ran.sce, *Ranunculus sceleratus*.

**Table 2:** Significance of the effects in ANOVA and variability coefficient (CV%) for the experiment.

Trait	Source of Variation			CV%
	Species	Ash Settling Ponds	Interaction	
Length of the stalks/grass blade [cm]	***	***	***	8.5
Number of the stalks/blade	***	***	***	13.6
Max. length of leaves [cm]	***	***	***	7.5
Max. length of flowers/panicles/inflorescences [cm]	***	***	***	12.7
Max. length of roots [cm]	***	ns	**	11.9

***, significant at *p* = 0.001; **, significant at *p* = 0.01; ns, non significant.

**Table 3:** Morphological features of the studied plant species growing on ash substrate (mean values ± standard error).

Trait	Species					
	Poa.pra	Agr.sto	Phr.aus	Jun.buf	Atr.pat	Ran.sce
Length of the stalk/plant blade [cm]	14.04 d ± 0.64	16.05 c ± 1.03	34.01 a ± 1.20	14.03 d ± 0.55	28.14 b ± 1.05	13.72 d ± 0.59
Number of the blade/ stalks	12.70 b ± 1.47	24.30 a ± 1.36	2.60 c ± 0.67	9.70 b ± 0.89	5.00 c ± 0.77	3.50 c ± 0.60
Max. length of leaves [cm]	7.90 b ± 0.31	5.40 c ± 0.43	15.00 a ± 0.44	8.80 b ± 0.44	3.30 c ± 0.15	4.50 c ± 0.27
Max. length of flower/panicle/inflorescence [cm]	9.70 b ± 0.54	–	–	6.00 c ± 0.60	26.00 a ± 1.32	5.80 c ± 0.47
Max. length of roots [cm]	13.20 c ± 0.61	14.70 c ± 0.45	10.90 d ± 0.53	29.00 a ± 1.04	20.00 b ± 0.76	16.80 cc ± 0.66

Poa.pra, *Poa pratensis*; Agr.sto, *Agrostis stolonifera*; Phr.aus, *Phragmites australis*; Jun.buf, *Juncus bufonius*; Atr.pat, *Atriplex patula*; Ran.sce, *Ranunculus sceleratus;* Mean values described by the same letter (a, b, c, d) are not different at 0.05 significance level; ±, standard error.

**Table 4:** The effect of ash settling ponds on estimated traits of plants (mean values ±standard error).

Trait	Ash Settling Ponds	
	I	II
Length of the stalks /grass blade [cm]	22.45 a ± 1.59	17.55 b ± 1.50
Number of the stalks / blade	11.97 a ± 1.98	7.30 a ± 1.00
Max. length of leaves [cm]	7.97 a ± 0.84	7.00 b ± 0.86
Max. length of flowers/panicles/inflorescences [cm]	8.97a ± 1.86	6.87 b ± 1.42
Max. length of roots [cm]	17.13 a ± 1.27	17.73 a ± 1.04

Mean values described by the same letter (a, b) are not different at 0.05 significance level; ±, standard error.

**Table 5:** Correlation coefficients and regression lines for relations between lenght of roots (x) and plant traits (y), n = 10.

Sp.	Trait (y)	Regression Line	Correlation Coefficient (r)	Determination Coefficient (r^2^ 100%)
Poa.pra	Length of stalk/the grass blade [cm]	y = 25.9 − 0.904x	−0.86 **	74.0
	Max. length of leaves [cm]	y = 3.11 + 0.363x	+0.70 *	49.0
Agr.sto	Number of the blade/ stalks	y = 109.6 − 5.810x	−0.77 **	59.3
Jun.buf	Max. length of leaves [cm]	y = 0.81 + 0.276x	+0.65 *	42.2
Ran.sce	Length of the grass blade/ stalk [cm]	y = 24.8 − 0.660x	−0.75 **	56.2
	Number of the blade/ stalks	y = 6.47 − 0.178x	−0.70 *	49.0

Sp., plant species; Poa.pra, *Poa pratensis*; Agr.sto, *Agrostis stolonifera*; Jun.buf, *Juncus bufonius*; Ran.sce, *Ranunculus sceleratus*; Level of significance 0.05 *; *p*, 0.01 **.

**Table 6:** Biological features of the identified plant species in the studied ash settling ponds (according to Plants atlas [7]).

Species	Flowering Period [Months]	Flowering Length [Weeks]	Adaptation Features to Bad Conditions	Life Form
Poa.pra	V-VIII	18	stoloniferous root systems, loose-tuft of grass, creeping rhizomes	perennial
Agr.sto	VI-VIII	13	stoloniferous root systems, produces a lot of vegetative shoots, numerous small seeds (0.07 mg)	perennial
Phr.aus	VI-IX	14	stoloniferous long, thick, blown root systems, long stem, large leaf area, production of numerous small seeds	perennial
Jun.buf	VI-IX	17	bundle root systems, numerous seeds (9380 seeds/m^2^)	annual
Atr.pat	VII-IX	14	top root systems with well-developed lateral roots; numerous seeds (up to 600 seeds)	annual
Ran.sce	V-IX	22	bundle root system; hollow stem, numerous seeds (up to 3000 seeds)	annual

Poa.pra, *Poa pratensis*; Agr.sto, *Agrostis stolonifera*; Jun.buf, *Juncus bufonius*; Ran.sce, *Ranunculus sceleratus*; V, May; VI, June; VII, July; VIII, August; IX, September.

**Table 7:** Total content of heavy metals in ash and plants (according to Gamrat et al. [4]).

Sample	Zn	Cu	Pb	Cr	Co	Ni
		mg·kg^-1^ Substrate				
Ash substrate	53.6	73.3	92.39	32.19	13.31	53.02
*Phragmites australis*	16.0	4.5	1.612	3.53	0.20	7.57
*Poa pratensis*	10.1	5.8	1.390	3.26	0.18	6.32
*Agrostis stolonifera*	24.6	8.0	0.664	4.86	0.82	8.16
*Juncus bufonius*	28.5	10.8	2.586	4.41	1.18	7.67
*Atriplex patula*	48.8	16.3	4.836	3.71	0.63	7.62

**Table 8:** Characteristics of selected climatic elements.

Months	Climatic Elements						
	Temperature [°C]			Precipitation			
	av.	max.	min.	av. [mm]	No of Days	Sum of Insolation [h]	Classification
January	3	12	−6	59	22	28	7
February	1	12	−9	4	8	111	1
March	6	18	−5	40	13	125	4
April	9	23	−2	29	10	226	3
May	13	26	2	48	14	209	4
June	17	29	4	33	12	191	2
July	19	34	8	62	19	222	4
August	21	36	4	15	8	312	1
September	14	29	3	34	15	186	3
October	9	21	−4	39	12	0	4
November	7	16	−2	49	22	39	5
December	7	15	−4	27	19	51	2

av., average; max., maximum; min., minimal.

## Data Availability

Not applicable.

## Abbreviations

Poa.pra: *Poa pratensis*; Agr.sto, *Agrostis stolonifera*; Phr.aus, *Phragmites australis*; Jun.buf, *Juncus bufonius*; Atr.pat, *Atriplex patula*; Ran.sce, *Ranunculus sceleratus*; Sp., plant species; C, control; P.S., private study; Stalks, stalks of plants /grass blade; Roots, roots/ stolons; Flowers, flowers/panicles/ inflorescences.
